# ChAdOx1 nCoV-19 (AZD1222) vaccine candidate significantly reduces SARS-CoV-2 shedding in ferrets

**DOI:** 10.1038/s41541-021-00315-6

**Published:** 2021-05-10

**Authors:** Glenn A. Marsh, Alexander J. McAuley, Gough G. Au, Sarah Riddell, Daniel Layton, Nagendrakumar B. Singanallur, Rachel Layton, Jean Payne, Peter A. Durr, Hannah Bender, Jennifer A. Barr, John Bingham, Victoria Boyd, Sheree Brown, Matthew P. Bruce, Kathie Burkett, Teresa Eastwood, Sarah Edwards, Tamara Gough, Kim Halpin, Jenni Harper, Clare Holmes, William S. J. Horman, Petrus Jansen van Vuren, Suzanne Lowther, Kate Maynard, Kristen D. McAuley, Matthew J. Neave, Timothy Poole, Christina Rootes, Brenton Rowe, Elisha Soldani, Vittoria Stevens, Cameron R. Stewart, Willy W. Suen, Mary Tachedjian, Shawn Todd, Lee Trinidad, Duane Walter, Naomi Watson, Trevor W. Drew, Sarah C. Gilbert, Teresa Lambe, S. S. Vasan

**Affiliations:** 1 CSIRO Australian Centre for Disease Preparedness, Geelong, VIC Australia; 2 Jenner Institute, Nuffield Department of Medicine, University of Oxford, Oxford, UK; 3 Department of Health Sciences, University of York, York, UK

**Keywords:** Viral infection, Vaccines, Viral infection

## Abstract

Vaccines against SARS-CoV-2 are likely to be critical in the management of the ongoing pandemic. A number of candidates are in Phase III human clinical trials, including ChAdOx1 nCoV-19 (AZD1222), a replication-deficient chimpanzee adenovirus-vectored vaccine candidate. In preclinical trials, the efficacy of ChAdOx1 nCoV-19 against SARS-CoV-2 challenge was evaluated in a ferret model of infection. Groups of ferrets received either prime-only or prime-boost administration of ChAdOx1 nCoV-19 via the intramuscular or intranasal route. All ChAdOx1 nCoV-19 administration combinations resulted in significant reductions in viral loads in nasal-wash and oral swab samples. No vaccine-associated adverse events were observed associated with the ChAdOx1 nCoV-19 candidate, with the data from this study suggesting it could be an effective and safe vaccine against COVID-19. Our study also indicates the potential for intranasal administration as a way to further improve the efficacy of this leading vaccine candidate.

## Introduction

In December 2019, an outbreak of the severe respiratory disease was first reported in Wuhan, China. The causative agent of this outbreak was rapidly identified as a novel coronavirus and denoted severe acute respiratory syndrome coronavirus-2 (SARS-CoV-2), responsible for COVID-19 disease^1–3^. In less than 12 months since its identification, SARS-CoV-2 has spread around the world and has been responsible for over 64 million infections and 1.5 million deaths worldwide as of December 2020^4^. Safe and effective vaccines and therapeutics will be essential for mitigating viral spread and for bringing the pandemic under control. Of the vaccines currently in development for COVID-19, the adenovirus vector-based candidate, ChAdOx1 nCoV-19 (AZD1222), is in late-stage clinical development, with Phase III trials in multiple countries currently underway^5^. As of January 2021, this vaccine has been approved by regulatory agencies in the UK and India for emergency use in humans^6^.

ChAdOx1 nCoV-19 is based upon the Jenner Institute’s adenovirus vaccine platform that utilises a replication-deficient Simian adenovirus vector engineered to express an antigen of interest^7^. This virus vector-based approach has been successfully used previously to generate several vaccine candidates against other infectious diseases including Middle-East respiratory syndrome coronavirus (MERS-CoV). A single dose of ChAdOx1 MERS was capable of protecting Rhesus macaques from infection and reducing MERS-CoV shedding in camels^8,9^. Phase I clinical trials of this vaccine candidate demonstrated the induction of robust antibody and T-cell responses^10^.

The ChAdOx1 nCoV-19 vaccine encodes the SARS-CoV-2 Spike protein, the main protein found on the surface of the virus, and after immunisation is subsequently expressed as an immunogen^11^. Administration of ChAdOx1 nCoV-19 to Rhesus macaques following a prime or prime-boost regimen induced a balanced humoral and cellular immune response. Following the challenge, vaccinated animals had a lack of pneumonia and a reduction in virus load in bronchioalveolar lavage fluid and the lower respiratory tract when compared to unvaccinated macaques^12^. Clinical trials have shown the vaccines to have an acceptable safety profile and are capable of inducing both humoral and cellular immune responses with recent data demonstrating efficacy^5,11^.

SARS-CoV-2 infection in ferrets most closely models asymptomatic or mild infection in humans. Infection of these animals is associated with the shedding of virus from the upper respiratory tract, with viral RNA detected in clinical samples taken up to day 9 post challenge^13–18^. The ferret model is considered to be a relevant animal model for testing SARS-CoV-2 vaccine candidate safety, immunogenicity, and efficacy by assessing the level of viral shedding and the determination of immune responses^19^.

We used the ferret model to assess the safety and efficacy of ChAdOx1 nCoV-19 against SARS-CoV-2 challenge. Ferrets received prime or prime-boost administrations of ChAdOx1 nCoV-19 as either an intramuscular injection or intranasal inoculation. Assessment of the immune response was performed post-vaccination, pre- and post challenge, and the ability to reduce viral shedding was also monitored.

## Results

### Immunogenicity of ChAdOx1 nCoV-19 in ferrets

**Fig. 1:**
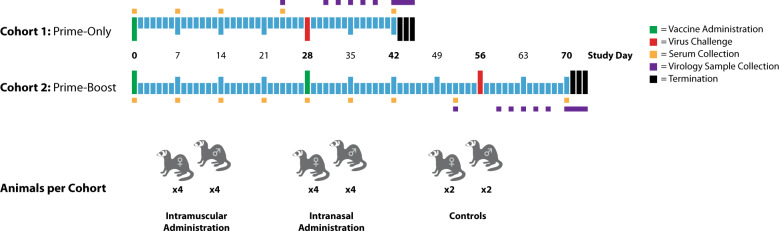
Study Outline. Groups of eight ferrets (four male, four female) received either a single (prime) or two (prime-boost) doses of ChAdOx1 nCoV-19 via the intramuscular or intranasal route. An additional four ferrets (two male, two female) were included as mock (PBS) vaccinated control animals. Samples were collected from all of the animals at the timepoints highlighted.

Eight animals per group (four male and four female) were administered ChAdOx1 nCoV-19 using a prime-only regimen (28-days pre-challenge) or a prime-boost regimen (56- and 28-days pre-challenge) either intramuscularly or intranasally with 2.5 × 10^10^ ChAdOx1 nCoV-19 virus particles per dose, which is half of the standard human dose (Fig. 1). As controls, four animals were administered phosphate-buffered saline (PBS) intramuscularly on days 56 and 28 pre-challenge (two male and two female) with an additional four animals (two male and two female) on day 28 pre-challenge. No adverse events or effects on the ferrets’ health were observed following ChAdOx1 nCoV-19 administration.

**Fig. 2:**
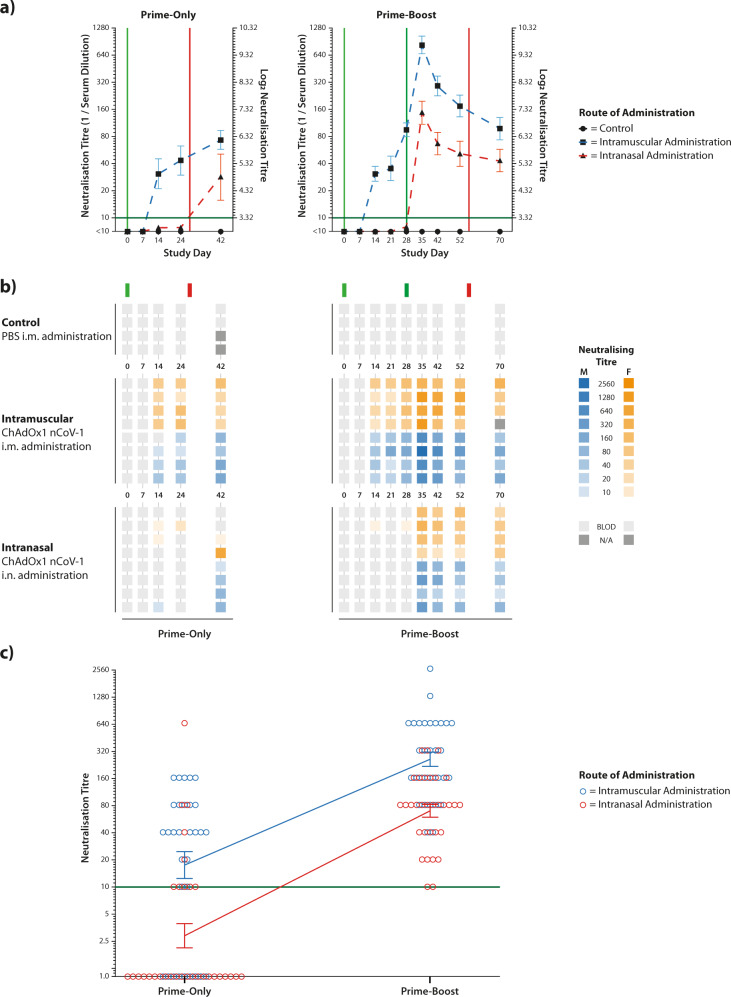
Serum virus-neutralisation titres against SARS-CoV-2. Individual ferret serum samples were tested using a virus microneutralisation assay with a twofold dilution series starting at 1:10, with the neutralisation titre stated as the highest dilution of serum leading to 100% virus neutralisation. **a** Mean titres were calculated for each study group from log_2_-transformed data and plotted with SEM. Green vertical lines represent dates of ChAdOx1 nCoV-19 administration, whilst red vertical lines represent virus challenge at study day 28 or 56 for the two cohorts, respectively. The dark green horizontal line represents the limit of detection of the assay. **b** Neutralising titres for individual ferrets were presented in a heatmap with vaccine administration and challenge dates represented with green and red lines, respectively. **c** Integrated analysis of neutralisation titres from prime-only and prime-boost administrations. Neutralisation titres from prime-only animals were combined from study days 7, 14, 24 and 40, whilst neutralisation titres from prime-boost animals were combined from study days 35, 42, 52 and 70. These represent the same timeframe relative to the challenge date. For both intramuscular and intranasal administration of ChAdOx1 nCoV-19, the increase in neutralisation titre from a boost dose is similar, as indicated by the almost-parallel linking lines. The vertical lines with error bars represent the mean and standard error of the mean (SEM) from log_2_-transformed data.

To measure antibody levels induced by the vaccine candidate, serum samples were collected on a weekly basis and neutralising antibody levels assessed using a standard virus microneutralisation assay. This assay assessed the ability of the samples to neutralise 100 TCID_50_ of SARS-CoV-2 virus. No virus-neutralising antibodies were detected in control animals (Fig. 2a, b).

Prime-only intramuscular administration of ChAdOx1 nCoV-19 reliably induced detectable neutralising antibodies prior to challenge, which was boosted by the challenge. Prime-only intranasal administration did not reliably induce detectable neutralising antibodies in the serum prior to challenge and, following challenge, only 75% of animals had measurable neutralising antibodies (Fig. 2b).

Administration of a boost dose of ChAdOx1 nCoV-19 by either route led to a sharp increase in neutralising antibody levels in all ferrets 7 days after the second vaccination, which then declined prior to the challenge. Integrated analysis of the neutralisation data demonstrated that the administration of a boost dose of ChAdOx1 nCoV-19 increased the overall neutralisation levels in a similar manner regardless of the administration route (Fig. 2c).

**Fig. 3:**
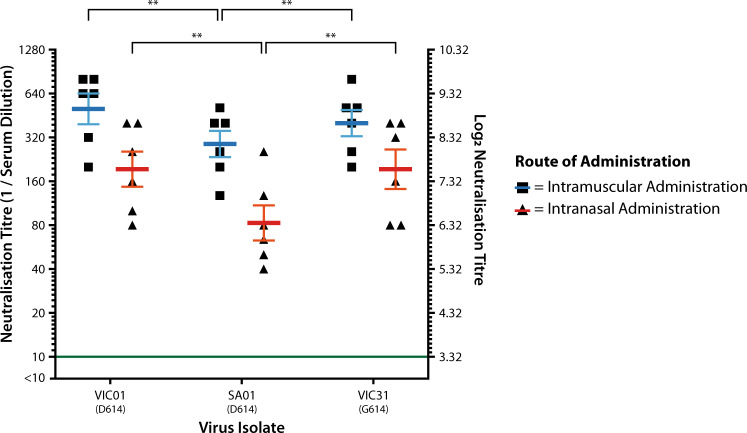
Comparative neutralisation of D614- and G614-containing isolates. Six serum samples from prime-boost animals were selected from study day 35 and 42 samples per administration route. Microneutralisation assays were performed for each sample using three Australian virus isolates containing D614 (VIC01, SA01) or G614 (VIC31) in the Spike protein. Triplicate titres were obtained for each serum/isolate combination, with log_2_-transformed mean values plotted as individual data points. Mean titres were calculated for each isolate/route of administration based upon log_2_-transformed data, which were plotted as blue or red lines, depending on the route of administration. Error bars represent SEM, **P < 0.01.

Consistent with McAuley et al.^24^, six serum samples per administration route were selected from available pre-challenge samples collected on study days 35 and 42 from the prime-boost animals. Each serum sample came from a different animal. Triplicate virus microneutralisation assays were performed with each serum sample against three Australian SARS-CoV-2 virus isolates: VIC01, SA01 and VIC31. Compared to the reference sequence for SARS-CoV-2 (Wuhan-Hu-1; NC_045512.2), these isolates have the following mutations in S: VIC01—S247R; SA01—no mutations; VIC31—D614G. Analysis of the results by route of administration revealed that neutralisation titres were greater in the animals that had received two doses of ChAdOx1 nCoV-19 via the intramuscular route (overall median titre 320) compared to those who received doses via the intranasal route (overall median titre 160; *P* < 0.001) (Fig. 3).

A mean neutralisation titre was calculated for each serum/isolate combination from the log_2_-transformed data. For animals that received intramuscular administration of ChAdOx1 nCoV-19, the overall mean neutralisation titres (blue bar) were within twofold of one another for each virus isolate, whilst for animals that received the intranasal administration, the overall mean neutralisation titre (red bar) was approximately 2.3-fold lower against the D614-containing SA01 isolate than the D614-containing VIC01 or the G614-containing VIC31 (Fig. 3). The mean neutralising titre against SA01 was significantly lower (*P* < 0.01) for each administration route. The reason for this variability is unclear, but we conclude that it is not directly due to the D614G mutation in the Spike protein given the similarity of neutralisation titres against VIC01 and VIC31.

**Fig. 4:**
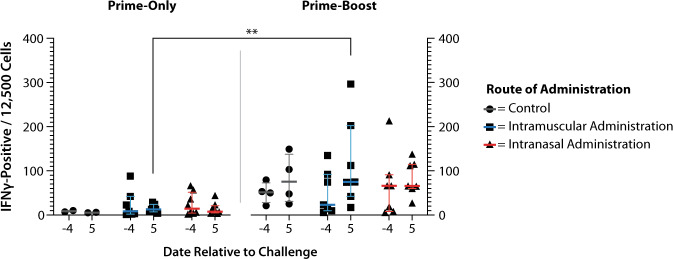
IFNγ-producing PBMC levels pre- and post challenge. PBMCs were isolated from blood samples taken on days −4 and 5, relative to challenge, for quantification of circulating IFNγ-producing cells by ELISpot assay. Prime-only animals had few IFNγ-producing cells, whilst prime-boost animals had greater numbers. Comparison of intramuscular prime-boost with prime-only post-challenge results demonstrated statistical significance (**P < 0.01). Horizontal lines represent mean and SEM.

Peripheral blood mononuclear cells (PBMCs) were isolated from the blood of ferrets on days −4 and 5, relative to virus challenge (study days 24 and 33 for prime-only animals; study days 52 and 61 for prime-boost animals). Due to an unexpected reaction at virus challenge, resulting in the euthanasia of two prime-only control ferrets (described in more detail below), values for only two prime-only control animals were obtained. Circulating interferon-gamma (IFNγ)-producing leukocytes were measured by IFNγ ELISpot (Fig. 4). None of the study groups had a significant change in IFNγ-positive PBMC levels between pre- and post-challenge samples. At day 5 post challenge, a significantly higher number of IFNγ-producing cells were observed in the intramuscular prime-boost group compared to the equivalent prime-only animals (*P* < 0.01). No other significant differences were observed.

### Clinical signs following challenge

No clinical signs attributable to viral infection were observed following intranasal challenge with 3 × 10^4^ TCID_50_ SARS-CoV-2 VIC01. None of the animals showed signs of fever (determined by subcutaneous transponder temperature readings) or weight loss, with ferrets remaining bright, alert and responsive throughout the course of the study. Haematological and clinical chemistry analysis of pre- and post-challenge samples revealed no values outside expected ranges. Importantly, no evidence of immune enhancement was observed in either the prime-only or prime-boost animals for either route of administration.

On day 28, immediately following the virus challenge of the prime-only animals, two control animals that received a single PBS placebo vaccination were euthanised due to a severe unexpected reaction. On day 56, during the challenge of prime-boost animals, acute unexpected reactions were observed in several animals, one of which was severe enough to warrant euthanasia. Affected ferrets showed varying degrees of respiratory distress, and necropsies were performed on euthanised animals. Histopathological assessment of respiratory tissues suggested an underlying allergic process. Root cause analysis identified the challenge inoculum as the triggering agent, with a commercial canine vaccine (Protech^®^ C3) administered to the ferrets as husbandry prophylaxis against canine distemper virus identified as the priming agent. Further analysis suggested an active immune response to a component(s) of FBS contained in both the C3 vaccine and the SARS-CoV-2 challenge inoculum^20^. The investigation found that the ChAdOx1 nCoV-19 vaccine candidate was not implicated in the unexpected reaction, and the observed reaction did not affect the study outcomes for the remaining ferrets.

### Virus shedding following challenge

Nasal wash, rectal and oral swab samples were collected at the prescribed time points of the study (Fig. 1). None of the samples collected pre-challenge (study day 24 for prime-only animals; day 52 for prime-boost animals) had detectable levels of SARS-CoV-2 E RNA, as determined by RT-qPCR.

**Fig. 5:**
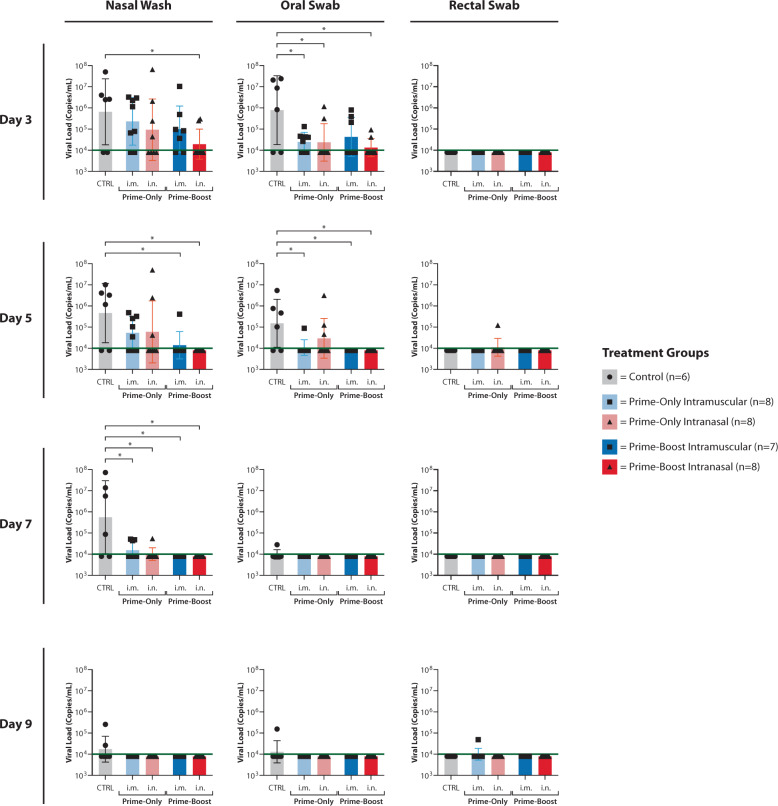
Viral load in nasal-wash and rectal and oral swab fluids as determined by RT-qPCR. RNA was extracted from samples collected on days 3, 5, 7 and 9 post challenge (study days 31, 33, 35 and 37 for prime-only animals; days 59, 61, 63 and 65 for prime-boost animals), and was analysed in duplicate using RT-qPCR assay detecting SARS-CoV-2 E RNA. Black points represent mean RNA copy number of the duplicate reactions for individual ferrets. The columns represent mean RNA copy numbers for each group, error bars represent SEM and the green lines are the limit-of-detection for the assay. Where no viral RNA was detected by RT-qPCR, a data point has been plotted at 3.9. Statistical analysis was performed by one-tailed *t* test, with statistical significance indicated by an asterisk (*P* < 0.05).

Viral RNA loads (log_10_ SARS-CoV-2 E copies/mL) for each sample collected post challenge are presented in Fig. 5 for days 3, 5, 7 and 9 post challenge. These time points correspond to study days 31, 33, 35 and 37 for prime-only animals, and study days 59, 61, 63 and 65 for prime-boost animals. No virus shedding was detected in any ferret samples collected on day 11 or 14 post challenge (study days 39 and 42 for prime-only animals; study days 67 and 70 for prime-boost animals; data not shown).

Compared to the control animals, average levels of virus shedding (as determined by RT-qPCR) in nasal-wash and oral swab samples were lower for each ChAdOx1 nCoV-19 Study Group. These reductions were statistically significant (*P* < 0.05) in nasal-wash samples on days 3, 5 and 7 post challenge, and oral swab samples on days 3 and 5 post challenge (Fig. 5). Administration of both prime and boost doses of ChAdOx1 nCoV-19 demonstrated a trend towards lower levels of shedding than prime-only administration, however, this trend was not statistically significant.

Virus isolation was attempted on PCR-positive samples, with only a single day 3 post-challenge nasal-wash sample from a prime-only, intranasal administration ferret positive for infectious virus. The infectious virus load in this sample was too low for a TCID_50_/mL titre to be calculated.

### Histopathological assessment of tissues

No evidence of viral-induced disease associated with the SARS-CoV-2 challenge was detected in any of the ferrets at the end of the trial. Moreover, no SARS-CoV-2 antigen labelling was detected by immunohistochemistry in the tissues examined (lung, spleen, tongue, nasal turbinate, pharynx, trachea, heart and retropharyngeal lymph nodes; data not shown as all negative). Minimal-to-moderate neutrophilic infiltrates were frequently present in the tracheal epithelium, bronchi, and terminal bronchioles of ferrets from all groups (including controls) and were considered incidental. Small numbers of ferrets had minimal-to-moderate eosinophilic infiltration of the tracheal mucosa and peribronchial interstitium. This change was present in animals from all groups and was unrelated to ChAdOx1 nCoV-19 administration. In animals euthanised immediately following unexpected reactions, eosinophilic airway inflammation was associated with pronounced mucosal oedema, implicating an acute allergic or anaphylactic response to the virus inoculum. No evidence of a reaction to either intramuscular or intranasal vaccine administration was observed in any animal.

## Discussion

Safe, effective and widely available vaccines will be instrumental in managing the ongoing SARS-CoV-2 pandemic. As of February 2021, there are over 240 COVID-19 vaccine candidates in development, with 63 undergoing clinical evaluation^21^. ChAdOx1 nCoV-19 has previously been tested in a rhesus macaque model of SARS-CoV-2 infection, where vaccination was demonstrated to prevent pneumonia but not nasal shedding of the virus^12^. The objective of this study was to use a second animal model of SARS-CoV-2 infection and further evaluate the safety and efficacy of ChAdOx1 nCoV-19. Importantly, this study was the first to propose and investigate an alternative COVID-19 vaccination route (intranasal) which is of relevance given SARS-CoV-2 is a respiratory pathogen, and the results from this study have led to subsequent human clinical trials for this candidate^22^.

All vaccine treatment groups presented statistically significant reductions in virus loads in nasal-wash and oral swab samples compared to control animals, demonstrating a vaccine-induced reduction in viral shedding. Animals that received prime-boost administration regimens had no statistically significant reductions in viral loads compared to animals that received prime-only administration of ChAdOx1 nCoV-19. This observation is in agreement with the previous studies investigating ChAdOx1 nCoV-19 efficacy in rhesus macaques^12^.

Neutralising antibody titres were generally higher in prime-boost animals and animals that received intramuscular administration of ChAdOx1 nCoV-19, which is to be expected as these studies measured serum neutralising antibody titres. Indeed, pre-challenge neutralising antibody titres were very similar between the prime-only animals and prime-boost animals that had received only a single dose. The Challenge of prime-only animals led to a boost-effect in neutralising antibodies, whilst this effect was not observed following the challenge of prime-boost animals. This would suggest that the third dose of ChAdOx1 nCoV-19 would likely not further-increase neutralising antibody levels, at least in the ferret model.

The lower serum neutralising titres measured in animals that received intranasally administered ChAdOx1 nCoV-19 compared to intramuscular administration can likely be explained through the induction of a more localised mucosal immune response compared to the systemic response induced by peripheral inoculation, as previously demonstrated with influenza vaccines^23^. Future studies (such as ref. ^22^) to look at the induction of mucosal immune responses following intranasal administration are strongly recommended, as only 25% (2/8) of animals that received prime-boost intranasal administration of ChAdOx1 nCoV-19 had detectable viral RNA in nasal-wash and oral swab samples on day 3 post challenge (compared to 75% (6/8) and 43% (3/7) positivity, respectively, in samples from prime-boost intramuscular-administered animals). Moreover, there no detectable viral RNA in any of the wash or swab samples for animals in the intranasal group on subsequent days.

The neutralising ability of vaccine-induced antibodies following intramuscular or intranasal prime-boost administration appeared not to be substantially affected by the D614G mutation that has arisen in the SARS-CoV-2 Spike protein. This is in agreement with studies looking at other vaccine candidates and post-challenge immune sera^24–26^. Whether vaccine efficacy will be affected by emerging mutations within neutralising epitopes in the receptor-binding domain remain to be determined.

ELISpot analysis of IFNγ-producing cells pre- and post challenge demonstrated that only the intramuscular prime-boost animals demonstrated a post-challenge increase compared to both prime-only groups, with no groups presenting significant change post challenge compared to pre-challenge levels.

Overall, ChAdOx1 nCoV-19 induced effective immune responses capable of reducing viral load in nasal-wash and oral swab samples. No unexpected reactions were observed following vaccination by either route. The results of this study complement and support parallel preclinical investigations in non-human primates and have supported regulatory applications for human clinical trials where preclinical evaluation in two animal models is expected^5,12^. The demonstration of a greater reduction in viral shedding following intranasal administration of ChAdOx1 nCoV-19 warrants further clinical investigation.

## Materials and methods

### Cells and viruses

The SARS-CoV-2 isolates hCoV-19/Australia/VIC01/2020 (VIC01), hCoV-19/Australia/SA01/2020 (SA01) and hCoV-19/Australia/VIC31/2020 (VIC31) were obtained as Passage 1 material from the Victorian Infectious Diseases Reference Laboratory (VIDRL; Melbourne, VIC, Australia)^27^. The SA01 isolate was made available courtesy of SA Pathology (Adelaide, SA, Australia). Virus propagation was performed in Vero E6 cells from BEI Resources (Manassas, VA, USA) for VIC01 and the European Collection of Animal Cell Cultures (ECACC; Porton Down, UK) for SA01 and VIC31, with cells grown in DMEM supplemented with 2% foetal bovine serum (FBS), 10 mM HEPES, 100 U/mL penicillin, 100 µg/mL streptomycin, and 250 ng/mL amphotericin B (all from Thermo Fisher Scientific; Scoresby, VIC, Australia). The virus stocks were characterised and demonstrated to have >99.9% sequence identity to the Wuhan-Hu-1 reference sequence (GenBank NC_045512.2), with no significant contaminants detected.

### Approval for animal studies

This study was reviewed and approved by the Animal Ethics Committee (AEC) at the CSIRO Australian Centre for Disease Preparedness (AEC 2004), in compliance with Australian national and state legislation pertaining to the use of animals in research. Challenge of animals with SARS-CoV-2 was performed under physical containment (PC)-4 conditions.

### Study design

This was a randomised, placebo-controlled study assessing ChAdOx1 nCoV-19 as a vaccine candidate against SARS-CoV-2 compared to a control (PBS). ChAdOx1 nCoV-19 was assessed by two different routes of administration, intranasal (i.n.) and intramuscular (i.m.). The study was carried out as a block design with two sex-matched cohorts of 20 outbred ferrets of ~4 months of age (total of 40 animals, 20 male and 20 female) that had previously received prime and boost doses of the canine C3 vaccine (Protech C3; Boehringer Ingelheim, Ingelheim am Rhein, Germany) as part of prescribed prophylaxis against canine distemper virus infection.

Prior to administration of ChAdOx1 nCoV-19, ferrets were implanted with a LifeChip Bio-Thermo transponder (Destron Fearing; Eagan, MN, USA). Subcutaneous temperature, rectal temperature and body weight was recorded for each animal, and pre-challenge serum samples were obtained.

On vaccination days, ChAdOx1 nCoV-19 stocks were diluted with sterile PBS to a concentration of 2.5 × 10^10^ virus particles per 100 µL. Sterile PBS was used as a placebo for unvaccinated control animals. The prepared material was then administered as either an intramuscular injection into the rear thigh muscle or dropwise to alternating nares, 100 µL per animal. Ferrets were monitored daily for 3 days post-administration for any reaction to ChAdOx1 nCoV-19. Following ChAdOx1 nCoV-19 administration, the ferrets were anaesthetised on a weekly basis to allow for the collection of serum samples to assess antibody responses (study days 0, 7, 14 and 24 for prime-only animals; study days 0, 7, 14, 21, 28, 35, 42 and 52 for prime-boost animals).

At challenge (study day 28 for prime-only animals; study day 56 for prime-boost animals), all animals were intranasally-exposed to a target dose of 3 × 10^4^ 50% Tissue Culture Infectious Dose (TCID_50_) of SARS-CoV-2 VIC01. Following the challenge, the ferrets were assessed at least once-per-day (twice daily for days 2–12 post challenge) for the presence of clinical signs, such as reduced interaction, fever (microchip temperature) and respiratory disease. On days 3, 5, 7, 9 and 11 post challenge (study days 31, 33, 35, 37 and 39 for prime-only animals; study days 59, 61, 63, 65 and 67 for prime-boost animals), ferrets were anaesthetised for collection of nasal wash, oral and rectal swab samples, as well as for the measurement of rectal temperature and body weight.

On study day 42 (for prime-only animals) or study day 70 (for prime-boost animals), ferrets were bled and had shedding samples collected, representative of day 14 post challenge. Over the following 3 days for each group of animals, ferrets were euthanised, and samples of the lung, spleen, tongue, nasal turbinate, pharynx, trachea, heart, and retropharyngeal lymph nodes were collected at necropsy for analysis.

### Determination of viral RNA load in samples

RNA was isolated from tissue, swab and nasal-wash samples using a MagMAX-96 Viral RNA Isolation Kit (Thermo Fisher Scientific) on a Kingfisher Flex instrument (Thermo Fisher Scientific). SARS-CoV-2 RNA load was determined by a quantitative real-time polymerase chain reaction (RT-qPCR), with each sample tested in duplicate using a modified assay targeting the viral E gene: forward primer 5′-AGTACGAACTTATGTACTCATTCGTT-3′; probe and reverse primer from Cormann et al.^28^. Copy numbers for individual samples were calculated using cycle threshold (*C*_T_) values as SARS-CoV-2 E gene copies per mL or per g, with an equation established from standard curve data using a synthetic DNA standard of known copy number.

### Virus-neutralisation assay

All serum samples were heat-inactivated (56 °C ± 2 °C for 30–35 min), then tested as a single replicate using a virus microneutralisation assay (VNT). Twofold dilutions of each serum sample were prepared in DMEM and 100 TCID_50_ SARS-CoV-2 VIC01 was added. After 1 h incubation at 37 °C/5% CO_2_, the virus:serum mixtures were combined with Vero E6 cells (ECACC) and returned to the incubator. At 3 days post-infection, cytopathogenic effects were assessed. The virus-neutralisation titre was expressed as the reciprocal value of the highest dilution of the serum that showed 100% inhibition of virus replication.

For the comparison of neutralisation titres against D614- and G614-containing SARS-CoV-2 isolates, six serum samples each were selected at random from the intramuscular and intranasal prime-boost animals collected at study day 35 or 42. No animal provided more than one serum sample. Each serum/isolate combination was tested in triplicate in a VNT as described above. A mean neutralisation titre was calculated for each of the combinations based upon the triplicate values using log_2_-transformed values.

### ELISpot assay

Blood was collected in lithium heparin tubes at time points pre- and post challenge (study days 24 and 33 for prime-only animals; study days 52 and 61 for prime-boost animals). Red blood cells were lysed using an ammonium chloride-based red blood cell lysis buffer. The remaining cells were washed with PBS and counted using a Miltenyi Scepter 2.0 with a 60-µm tip (Miltenyi Biotech; Bergisch Gladbach, Germany). Ferret IFNγ ELISpot plates (Mabtech; Stockholm, Sweden) were prepared following the manufacturer’s instructions. Briefly, plates were washed with sterile PBS five times and 200 µL DMEM containing 10% FBS was added to the required wells. The plates were incubated at 37 °C/5% CO_2_ for 30 min, followed by removal of the medium and addition of 200 µL cell suspension (1.25 × 10^4^ cells/well) to each of the required wells. The plate was then sealed and incubated at 37 °C for 24 h. At the end of the incubation, cells were removed and the plates were washed five times with 200 µL sterile PBS. Detection antibody and streptavidin-ALP were separately diluted 1:1000 in PBS with 0.05% FBS. In total, 100 µL detection antibody was added to each well and was incubated at room temperature for 2 h. The samples were washed five times with 200 µL sterile PBS, and 100 µL streptavidin-ALP was added per well. The plate was incubated at room temperature for 1 h. At the end of the incubation, the samples were again washed five times with 200 µL sterile PBS per well, and 100 µL substrate solution was added. The plate was incubated at room temperature until distinct spots emerged on the membrane. Once spots were clear, the plates were washed extensively with tap water to remove the substrate solution and halt the reaction. The plate was left to dry at room temperature for 30 min before being read using an AID vSPOT Spectrum ELISpot Reader (Autoimmun Diagnostika; Straßberg, Germany).

### Histology

Samples for histological and immunohistochemical analysis were collected into 10% neutral-buffered formalin and processed by routine histological methods. Tissue sections of 4 µm were stained with haematoxylin and eosin for histological assessment. On consecutive sections, antigen retrieval for immunohistochemistry was performed using the Dako PT Link (Agilent Technologies; Glostrup, Denmark) and Dako EnVision FLEX Target retrieval solution, pH 9 (Agilent Technologies). For retrieval, tissues were heated at 97 °C for 20 min, followed by cooling to 65 °C. Endogenous peroxidases were quenched using a 3% H_2_O_2_ solution. The sections were then incubated for 60 min with a polyclonal rabbit antibody against SARS-CoV-2 nucleocapsid protein (Sino Biological; Beijing, China) at a 1:8000 dilution, then with EnVision FLEX horseradish peroxidase-labelled secondary antibody and 3-amino-9-ethylcabazole (AEC) chromogen (Agilent Technologies). Sections were rinsed between each step with Tris buffer, pH 7.6. Slides were counterstained with Lillie-Mayer haematoxylin.

### Statistical analysis

To assess the effect of the D614G mutation and the two vaccine administration routes on the log_2_ neutralisation titre, a two-way mixed-effects analysis of variance (ANOVA) was performed, with the three test replicates being treated as a random effect. For the post hoc analysis to detect significant factor-level differences, pairwise comparisons were performed using Tukey’s adjustment. These analyses were performed in R 4.0, using the *nlme v3.1* package for the mixed-effects ANOVA modelling, and *multicomp v1.4* for post hoc comparisons.

Analysis of statistical significance in the ELISpot and viral shedding data was performed using Student’s *T* test in GraphPad Prism v9.0.0.

### Reporting summary

Further information on research design is available in the Nature Research Reporting Summary linked to this article.

## Supplementary information

Reporting Summary

## Data Availability

The data that support the findings of this study are available from the corresponding author upon reasonable request.
