# Supplementary material for: ChAdOx1 nCoV-19 (AZD1222) vaccine candidate significantly reduces SARS-CoV-2 shedding in ferrets
Source: NPJ Vaccines. 2021 May 10;6:67. doi: 10.1038/s41541-021-00315-6 (PMC8110954; doi:10.1038/s41541-021-00315-6)
Supplement: Supplementary file 1 — Reporting Summary [file 41541_2021_315_MOESM1_ESM.pdf]

nature research

Corresponding author(s): *Double-blind peer review submissions: write DBPR and your manuscript number here instead of author names.*

Last updated by author(s): *YYYY-MM-DD*

Reporting Summary

Nature Research wishes to improve the reproducibility of the work that we publish. This form provides structure for consistency and transparency in reporting. For further information on Nature Research policies, see our Editorial Policies and the Editorial Policy Checklist.

Statistics

For all statistical analyses, confirm that the following items are present in the figure legend, table legend, main text, or Methods section.

n/a	Confirmed	
☐	☒	The exact sample size (*n*) for each experimental group/condition, given as a discrete number and unit of measurement
☐	☒	A statement on whether measurements were taken from distinct samples or whether the same sample was measured repeatedly
☐	☒	The statistical test(s) used AND whether they are one- or two-sided<br>*Only common tests should be described solely by name; describe more complex techniques in the Methods section.*
☐	☒	A description of all covariates tested
☐	☒	A description of any assumptions or corrections, such as tests of normality and adjustment for multiple comparisons
☐	☒	A full description of the statistical parameters including central tendency (e.g. means) or other basic estimates (e.g. regression coefficient) AND variation (e.g. standard deviation) or associated estimates of uncertainty (e.g. confidence intervals)
☐	☒	For null hypothesis testing, the test statistic (e.g. *F*, *t*, *r*) with confidence intervals, effect sizes, degrees of freedom and *P* value noted<br>*Give P values as exact values whenever suitable.*
☒	☐	For Bayesian analysis, information on the choice of priors and Markov chain Monte Carlo settings
☒	☐	For hierarchical and complex designs, identification of the appropriate level for tests and full reporting of outcomes
☒	☐	Estimates of effect sizes (e.g. Cohen's *d*, Pearson's *r*), indicating how they were calculated

Our web collection on statistics for biologists contains articles on many of the points above.

Software and code

Policy information about availability of computer code

Data collection	Data was collected on paper records and transcribed into Microsoft Excel
Data analysis	Analyses were performed in R 4.0, using the nlme v3.1 package for the mixed effects ANOVA modelling, and multicomp v1.4 for post hoc comparisons. Student's t-test was performed in GraphPad Prism v9.0.0.

For manuscripts utilizing custom algorithms or software that are central to the research but not yet described in published literature, software must be made available to editors and reviewers. We strongly encourage code deposition in a community repository (e.g. GitHub). See the Nature Research guidelines for submitting code & software for further information.

Data

Policy information about availability of data

All manuscripts must include a data availability statement. This statement should provide the following information, where applicable:

- Accession codes, unique identifiers, or web links for publicly available datasets
- A list of figures that have associated raw data
- A description of any restrictions on data availability

The data that support the findings of this study are available from the corresponding author upon reasonable request.

Field-specific reporting

Please select the one below that is the best fit for your research. If you are not sure, read the appropriate sections before making your selection.

☒ Life sciences ☐ Behavioural & social sciences ☐ Ecological, evolutionary & environmental sciences

For a reference copy of the document with all sections, see nature.com/documents/nr-reporting-summary-flat.pdf

Life sciences study design

All studies must disclose on these points even when the disclosure is negative.

Sample size	n=8 (4 male and 4 female) ferrets were used for each treatment group. Number of animals selected based upon statistical advice.
Data exclusions	Data was not excluded, although data from two control animals and one test animal was no available due to early euthanasia of the animals.
Replication	Duplicate RT-qPCR assays were run for each virology sample, with mean value reported. Neutralisation titres in the D614G neutralisation study were obtained in technical triplicate with mean of log2-transformed data reported.
Randomization	Animals were randomised into control and test groups per internal SOP.
Blinding	Laboratory personnel remained blinded to group assignments of the animals during the course of the laboratory work. Unblinding was performed prior to statistical analysis and interpretation of the data. Blinding was not employed for the D614G neutralisation study.

Reporting for specific materials, systems and methods

We require information from authors about some types of materials, experimental systems and methods used in many studies. Here, indicate whether each material, system or method listed is relevant to your study. If you are not sure if a list item applies to your research, read the appropriate section before selecting a response.

Materials & experimental systems

n/a	Involved in the study	
☐	☒	Antibodies
☐	☒	Eukaryotic cell lines
☒	☐	Palaeontology and archaeology
☐	☒	Animals and other organisms
☒	☐	Human research participants
☒	☐	Clinical data
☒	☐	Dual use research of concern

Methods

n/a	Involved in the study	
☒	☐	ChIP-seq
☒	☐	Flow cytometry
☒	☐	MRI-based neuroimaging

Antibodies

Antibodies used	Rabbit polyclonal antibody against SARS-CoV-2 nucleocapsid protein (Cat No. 40588; Sino Biological, Beijing, China).
Validation	Antibody validated in-house for detection of SARS-CoV-2 antigen in tissue sections.

Eukaryotic cell lines

Policy information about cell lines

Cell line source(s)	Vero E6 cells: European Collection of Animal Cell Cultures (via Sigma Aldirch)
Authentication	Cell stocks 5 passages from receipt
Mycoplasma contamination	Not detected by qPCR
Commonly misidentified lines (See ICLAC register)	N/A

Animals and other organisms

Policy information about studies involving animals; ARRIVE guidelines recommended for reporting animal research

Laboratory animals	Domestic ferret (Mustela putorius furo), sex matched, approximately 4 months of age.
Wild animals	Study did not use wild-caught animals
Field-collected samples	Study did not use samples collected from the field
Ethics oversight	Studies approved under ACDP AEC2004

Note that full information on the approval of the study protocol must also be provided in the manuscript.
